# Detection in the United Kingdom of the *Neisseria gonorrhoeae* FC428 clone, with ceftriaxone resistance and intermediate resistance to azithromycin, October to December 2018

**DOI:** 10.2807/1560-7917.ES.2019.24.10.1900147

**Published:** 2019-03-07

**Authors:** David W Eyre, Katy Town, Teresa Street, Leanne Barker, Nicholas Sanderson, Michelle J Cole, Hamish Mohammed, Rachel Pitt, Maya Gobin, Charles Irish, Daniel Gardiner, James Sedgwick, Charles Beck, John Saunders, Deborah Turbitt, Clare Cook, Nick Phin, Bavithra Nathan, Paddy Horner, Helen Fifer

**Affiliations:** 1 Big Data Institute, University of Oxford, Oxford, United Kingdom; 2 Nuffield Department of Medicine, University of Oxford, Oxford, United Kingdom; 3 National Incident Management Team, Public Health England, London, United Kingdom; 4 Population Health Sciences, University of Bristol, Bristol, United Kingdom; 5 These authors contributed equally to this work

**Keywords:** gonorrhoeae, treatment failure, antimicrobial resistance, antimicrobial treatment, whole-genome sequencing

## Abstract

We describe detection in the United Kingdom (UK) of the drug-resistant *Neisseria gonorrhoeae* FC428 clone, with ceftriaxone resistance and intermediate azithromycin resistance. Two female patients developed infection following contact with UK-resident men from the same sexual network linked to travel to Ibiza, Spain. One case failed treatment with ceftriaxone, and azithromycin and gentamicin, before successful treatment with ertapenem. Both isolates had indistinguishable whole-genome sequences. Urgent action is essential to contain this drug-resistant strain.

Antimicrobial resistance in *Neisseria gonorrhoeae* is a major public health concern, in particular resistance to ceftriaxone which is the last available drug for empirical monotherapy for gonorrhoea. Here, we report further international spread of the *N. gonorrhoeae* FC428 clone associated with ceftriaxone resistance and intermediate resistance to azithromycin.

The FC428 clone was first isolated in Japan in January 2015 [1], with a second isolate, FC460, obtained from the same patient [2]. This clone was subsequently reported in 2017 in Australia (A7536, A7846) [3], Canada (47707) [4], Denmark (GK124) [5] and France (F90) [6], and in 2018 in Ireland (IR72) [7]. However despite this global dissemination, sustained local transmission has not been reported to date. We describe a transmission cluster that probably involved at least four people resident in the United Kingdom (UK). We report the clinical management, microbiology and whole-genome sequencing findings.

## Case descriptions

Case 1, a female patient, presented to a sexual health clinic in the UK in October 2018 with urinary symptoms. She had had unprotected vaginal intercourse with more than one male partner, 2 months before presentation, while on holiday in Ibiza, Spain. These partners were normally resident in the UK. The patient had a positive *N. gonorrhoeae* nucleic acid amplification test (NAAT) and endocervical culture. Before the results of susceptibility testing were available, she was treated empirically, in line with national treatment guidelines at the time, with a single dose of intramuscular ceftriaxone 500 mg and oral azithromycin 1 g. A NAAT test of cure (TOC) 2 weeks after treatment was negative. We were unable to trace her partners.

Case 2, a female patient, presented to another sexual health clinic elsewhere in the UK in November 2018 for an asymptomatic sexual health screen. Two weeks previously, she had had unprotected vaginal, oral and anal sex with an asymptomatic UK-resident man who had been in Ibiza and had links with the same sexual network as Case 1. She had positive vaginal and rectal *N. gonorrhoeae* NAAT results, a negative throat *N. gonorrhoeae* NAAT and was culture-negative at all three sites. She was treated empirically 1 week later with a single dose of intramuscular ceftriaxone 1 g and at this visit reported rectal symptoms. She initially responded clinically but her symptoms relapsed, and she was culture-positive on a vaginal sample taken 10 days later by her general practitioner, and culture and NAAT-positive at both the rectum and urogenital tract but not the throat when she attended the sexual health clinic a further 4 days later for treatment. She failed subsequent treatment with single dose intramuscular gentamicin 240 mg plus oral azithromycin 2 g but cleared the infection with intravenous ertapenem 1 g once daily for 3 days. A NAAT TOC taken 2 weeks later was negative at both sites.

The male sexual contact of Case 2 tested negative for gonorrhoea on urine NAAT testing in November 2018 without treatment and he did not report symptoms before or after sexual intercourse with Case 2. However, given his link to the same sexual network as Case 1, he is likely to have been the source of Case 2’s infection, and to have spontaneously cleared his infection [8]. Further information on other partners of Case 2’s male contact was not available. Case 2 also had unprotected oral sex and protected vaginal sex with a new male partner 8 days after receiving the first treatment (ceftriaxone), before her symptoms relapsed. This partner subsequently tested *N. gonorrhoeae* NAAT-positive at the pharynx but had a negative pharyngeal culture and negative urine NAAT. Although this case may have had pre-existing asymptomatic pharyngeal carriage, we considered that his positive test was possibly explained by acquisition of *N. gonorrhoeae* from Case 2. He was therefore also treated with intravenous ertapenem 1 g once daily for 3 days and had a negative TOC 2 weeks later.

## Phenotypic characterisation

One *N. gonorrhoeae* isolate from each culture-positive case (Case 1: H18-209 and Case 2: H18-502) was sent to Public Health England’s national reference laboratory for characterisation. Antimicrobial susceptibility testing was undertaken using minimum inhibitory concentration (MIC) gradient diffusion strips. Results were interpreted using European Committee on Antimicrobial Susceptibility Testing (EUCAST) breakpoints [9].

Both isolates showed resistance to ceftriaxone (MIC 1.0 mg/L), cefixime (MIC 2.0 mg/L), penicillin (MIC 2.0 mg/L), tetracycline (MIC 2.0 mg/L) and ciprofloxacin (MIC > 32 mg/L), and intermediate resistance to azithromycin (MIC 0.5 mg/L). The isolates were susceptible to spectinomycin (MIC 8.0 mg/L) and did not produce a β-lactamase. MICs for gentamicin and ertapenem were 4.0 mg/L and 0.032 mg/L, respectively, which represent low values likely to be susceptible although no formal breakpoints are defined.

## Genomic characterisation

Whole genome sequencing (WGS) was undertaken using Illumina MiSeq and Oxford Nanopore Technologies (ONT) sequencing as previously described [10]. Raw sequence reads are available under NCBI BioProject PRJNA523794. Maximum likelihood phylogenies [11], based on mapping to the NCCP11945 reference genome, and corrected for recombination [12], were used to compare the sequences of the cases’ isolates to previously described ceftriaxone-resistant isolates [1-7,10,13-15].

Antimicrobial resistance determinants were identified from ONT and Illumina data as described previously, using a combination of de novo assembly and mapping-based approaches [16]. BEAST v1.10.4 [17] was used to generate time-dated phylogenies using a recombination-corrected whole genome alignment and a Hasegawa-Kishino-Yano (HKY) substitution model. A strict molecular clock was fitted. As there were a limited number of genomes, the prior distribution for the clock rate was chosen to match the credibility interval for the *N. gonorrhoeae* mutation rate determined previously [18]. Outputs from three independent chains were merged after checking for convergence and a sufficient effective sample size.

The two isolates’ genomes were indistinguishable and were from multilocus sequence typing (https://pubmlst.org/neisseria) ST-1903, *N. gonorrhoeae* multi-antigen sequence typing (NG-MAST; http://www.ng-mast.net) ST-1614 (*porB* 1053, *tbpB* 33) and *N. gonorrhoeae* sequence typing for antimicrobial resistance (NG-STAR; https://ngstar.canada.ca) ST233. The *penA* allele was identical to that seen in FC428 (*penA*-60.001), containing two key ceftriaxone resistance mutations, A311V and T483S, as well as G545S, I312M and V316T. There were no 23S rRNA mutations; azithromycin intermediate resistance was conferred by an *mtrR* promoter deletion (−35A) and ciprofloxacin resistance by *parC* S87R and *gyrA* S91F and D95A substitutions.

**Figure 1:**
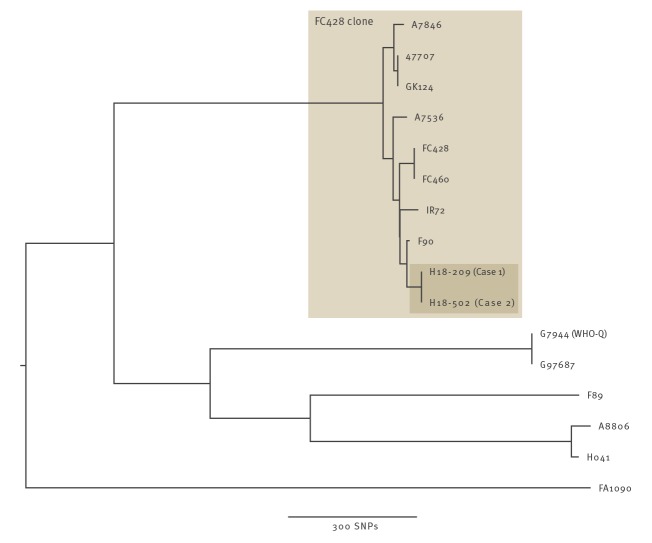
Ceftriaxone-resistant *Neisseria gonorrhoeae* isolates (n=15), including from Case 1 and Case 2, United Kingdom, 2018

Comparison with previously sequenced ceftriaxone-resistant isolates demonstrated the cases’ isolates were from the FC428 clone (Figure 1).

SNP: single nucleotide polymorphism.

Maximum likelihood phylogeny, based on mapping to the NCCP11945 reference genome, corrected for recombination. Previously described ceftriaxone-resistant isolates were used for comparison [1-7,10,13-15]. All isolates shown were resistant to ceftriaxone, except FA1090, which was included to root the tree.

**Figure 2:**
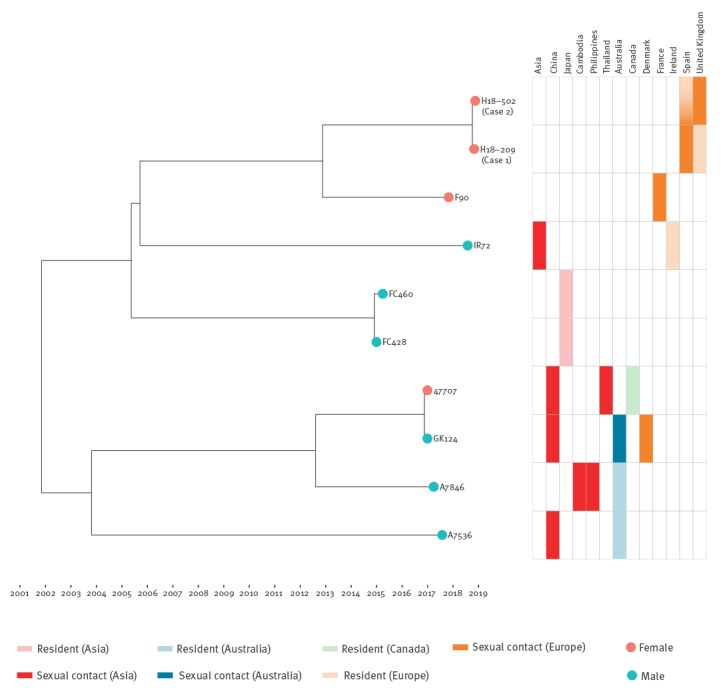
Time-dated Bayesian phylogeny of *Neisseria gonorrhoeae* FC428 clone cases and epidemiological details, 2015–2019 (n = 10)

A time-dated Bayesian phylogeny of all available sequences from the FC428 clone is shown in Figure 2, with published details of the location of each case’s country of residence and sexual contacts. The current cases’ genomes are most closely related to F90; both the present culture-positive patients and the F90 patient had not travelled outside of Europe; their sexual contacts were, respectively, in Spain (with men from the UK) and in France. The remainder of the cases were diagnosed globally (Ireland, Japan, Canada, Denmark, Australia), however, all had had sexual contacts in Asia.

The right-hand panel indicates the country of residence and any links to sexual contacts in other countries. Cases with a sexual contact in their country of residence are shown with that country coloured on the basis of the sexual contact. The box indicating Spain for H18-502 is shown partially shaded to reflect Case 2’s partner’s sexual contact in Spain.

Within the FC428 clone, after correction for recombination, 267 unique single nucleotide polymorphisms (SNPs) were identified among the genomes. Such diversity makes direct transmission between any of the previously described cases unlikely, with the exception of GK124 and 47707 which were genetically indistinguishable. To our knowledge, this link has not been previously reported. These isolates from Denmark and Canada were from a man and a woman who both reported sexual contact in China with a member of the opposite sex. 

Based on previously determined rates of *N. gonorrhoeae* evolution, the date of the most recent common ancestor of the FC428 clone was estimated as October 2001 (95% highest posterior density interval: May 1999 to March 2004). This is considerably before the first reported case from the clone, FC428, in January 2015. This suggests the clone emerged and persisted in a setting where antimicrobial susceptibility testing for *N. gonorrhoeae* is not routinely undertaken. This allowed it to remain undetected for probably more than 10 years and enabled the emergence of diversity in the infection reservoir with which each of the reported cases has separately come into contact.

## Discussion

There is growing evidence that the FC428 clone has the potential to spread globally, which is of concern given it is resistant and intermediate resistant to the only two remaining empirical treatment options for *N. gonorrhoeae*, ceftriaxone and azithromycin. Although reported new cases are uncommon and mostly described following sexual contact in South East Asia, our cases, with the previously reported F90 case, provide evidence of transmission occurring within Europe. As the transmission between our cases is likely to have occurred between UK residents visiting Ibiza, a well-known European party destination, there is a risk that further undetected transmission has occurred.

The location of the sexual contacts of the remaining cases described within the clone, suggests South East Asia or China, is the likely reservoir for this clone. There is therefore an urgent need for improved access in the region to *N. gonorrhoeae* diagnostics and antimicrobial resistance detection [19]. This is required for surveillance so that the burden of antimicrobial resistance can be better quantified and rational antibiotic treatment guidelines made. At the level of the individual patient, it is needed to prevent onward transmission of drug-resistant gonorrhoea.

We report a *N. gonorrhoeae* transmission cluster involving the FC428 clone with two confirmed female cases and at least two probable additional infections in the male partners of the cases. Given that the partners of Case 1 could not be contacted and that it is unclear how long the asymptomatic male contact of Case 2 was infected [8,20] and unclear if this *N. gonorrhoeae* strain was initially acquired by UK residents before or during travel to Ibiza, it is likely that there has been onward transmission from one or more undetected cases. As a result, Public Health England have introduced enhanced monitoring of data submitted by local laboratories on *N. gonorrhoeae* antimicrobial resistance to ensure all ceftriaxone-resistant isolates identified are sent to Public Health England for confirmation and investigation promptly to help reduce further spread.

Case 2 required treatment with 3 days of intravenous ertapenem after failed treatment with ceftriaxone, and with azithromycin and gentamicin. However, widespread use of ertapenem may pose logistical difficulties where sexual health services are not set up to administer intravenous therapy on consecutive days. In addition, use of carbapenems may promote drug resistance in other organisms, including Enterobacteriaceae, where carbapenems are usually reserved for multidrug-resistant infections. Therefore, new treatments for *N. gonorrhoeae* infection are urgently required. Zoliflodacin is a novel antibiotic active against urogenital infections, but with only limited efficacy in pharyngeal infections [21]. Several other potential agents are under development [22].

European public health agencies and sexual health clinicians should be aware that the FC428 clone has potential to spread in Europe. This threatens the effectiveness of gonorrhoea treatment. New treatments are required that are effective against all infected sites and can be delivered by existing sexual health infrastructure. Ongoing surveillance of antimicrobial resistance with culture or emerging molecular methods [23], test of cure, extra-genital sampling and effective partner notification are vital to maintain control of *N. gonorrhoeae*.
